# Impact of nonphysician providers on spatial accessibility to primary care in Iowa

**DOI:** 10.1111/1475-6773.13280

**Published:** 2020-02-26

**Authors:** Sean G. Young, Thomas S. Gruca, Gregory C. Nelson

**Affiliations:** ^1^ Department of Environmental and Occupational Health University of Arkansas for Medical Sciences Little Rock Arkansas; ^2^ Tippie College of Business University of Iowa Iowa City Iowa; ^3^ Office of Statewide Clinical Education Programs Carver College of Medicine University of Iowa Iowa City Iowa

**Keywords:** advance nurse practitioners, physician assistants, primary care, spatial access

## Abstract

**Objective:**

To assess the impact of nonphysician providers on measures of spatial access to primary care in Iowa, a state where physician assistants and advanced practice registered nurses are considered primary care providers.

**Data Sources:**

2017 Iowa Health Professions Inventory (Carver College of Medicine), and minor civil division (MCD) level population data for Iowa from the American Community Survey.

**Study Design:**

We used a constrained optimization model to probabilistically allocate patient populations to nearby (within a 30‐minute drive) primary care providers. We compared the results (across 10 000 scenarios) using only primary care physicians with those including nonphysician providers (NPPs). We analyze results by rurality and compare findings with current health professional shortage areas.

**Data Collection/Extraction Methods:**

Physicians and NPPs practicing in primary care in 2017 were extracted from the Iowa Health Professions Inventory.

**Principal Findings:**

Considering only primary care physicians, the average unallocated population for primary care was 222 109 (7 percent of Iowa's population). Most of the unallocated population (86 percent) was in rural areas with low population density (< 50/square mile). The addition of NPPs to the primary care workforce reduced unallocated population by 65 percent to 78 252 (2.5 percent of Iowa's population). Despite the majority of NPPs being located in urban areas, most of the improvement in spatial accessibility (78 percent) is associated with sparsely populated rural areas.

**Conclusions:**

The inclusion of nonphysician providers greatly reduces but does not eliminate all areas of inadequate spatial access to primary care.

**What this study adds**

- Many locations in Iowa with sufficient spatial access to primary care are considered health professional shortage areas, while many locations with poor access are excluded from any shortage designation whatsoever.
- Including NPPs results in a considerable improvement in spatial access to primary care, particularly in rural areas.
- Areas of poor spatial accessibility to primary care persist even after including NPPs, which are not reflected by currently used shortage designations.

## 1 INTRODUCTION

The aging of the US population, the obesity epidemic, and insurance reforms are increasing the demand for primary care services.^1^, ^2^ At the same time, the number of adult primary care physicians is not expected to grow at a pace sufficient to meet this expected increase.^3^, ^4^ High levels of professional burnout among family medicine physicians^5^ and an increasing number of primary care doctors transitioning to part‐time roles^6^ may reduce the realized availability of individual primary care physicians. This combination of circumstances has raised alarms about a worsening of the current shortage of primary care physicians. While estimates vary, the United States is expected to have a shortage of 21 100 to 55 200 primary care physicians in the next decade.^1^, ^4^, ^6^, ^7^, ^8^

Several solutions have been proposed to help meet the projected demand for primary care services, including expanding the roles of nonphysician providers (NPPs) such as advanced registered nurse practitioners (APRNs) and physician assistants (PAs) in primary care.^9^ Reflecting the potential impact of NPPs on access to primary care, the Health Resources and Services Administration (HRSA) suggests that NPPs be included in the determination of shortage areas for primary care.^8^ Studies outside the United States have demonstrated the important contribution of NPPs to primary care accessibility.^10^, ^11^ However, to our knowledge, the effects of NPPs on the spatial accessibility of primary care have not been modeled at the local level in the United States. As noted in a national report^8^ from the National Center for Health Workforce Analysis, “(m)ore detailed analysis of the adequacy of the supply within local geographic areas…is needed to better understand the adequacy of the primary care workforce.”

An individual's access to care is determined by three distinct steps^12^, ^13^: (a) gaining access to the system, usually via insurance coverage, (b) physical access to a location where necessary medical care is provided (geographic access), and (c) establishing a trusting relationship with a health care provider, which requires available provider capacity.^14^ Without such capacity, the expansion of insurance coverage, for example, would not result in increased access to primary care. Despite changes to insurance coverage in recent years, disparities in access to care continue with respect to age, sex, race, ethnicity, socioeconomic status, language, disability status, sexual orientation, gender identity, and residential location (metropolitan area versus nonmetropolitan area).^15^ Of these groups with persistent access barriers, we focus on rural patients in Iowa. Our analysis considers two key components of access: geographic access and provider capacity. The combination of geographic access and provider capacity is known as spatial access.^16^

To assess the impact of NPPs on spatial access to primary care requires detailed data on individual APRNs and PAs. The extant state‐level projections^17^ are inadequate for the task. For example, to estimate the number of PAs in primary care, HRSA's model uses a national average of 40 percent. However, this figure actually varies widely from 16 to 58 percent.^18^ In addition, there seems to be a great deal of confusion regarding the training of APRNs (eg, family nurse practitioner) versus their practice specialty (primary care versus specialty care). For example, in North Carolina, the proportion of APRNs trained in primary care fields was nearly 80 percent, while only 58 percent were actually practicing in primary care.^19^

A second challenge is acquiring detailed and accurate information on individual provider locations to determine spatial accessibility.^16^ Often the AMA's Physician Masterfile is used for such analyses. However, a substantial portion of AMA records lack office addresses (generally 10 to 25 percent) and the use of physician mailing addresses leads to systematic geocoding errors and bias in accessibility studies.^20^, ^21^ For nonphysicians, such data on practice locations are even harder to come by. The current state‐level model^17^ only considers physician availability, measured using population‐to‐provider ratios. The second aspect—geographic access—is measured using travel times or distances between patients and providers. Geographic access should be included in assessing the impact of NPPs since it is crucial in the determination of health professional shortage areas (HPSAs) for primary care.^22^

A third issue involves the measurement of spatial access to primary care. Most attempts to measure spatial accessibility have relied on gravity models, such as the two‐step floating catchment area (2SFCA) method and its many variations.^23^, ^24^, ^25^ However, these methods suffer from numerous drawbacks, including double‐counting. This bias would overestimate demand in densely populated areas or overestimate supply in areas with many providers.^26^

In contrast, optimization models offer a more advanced and flexible approach with several advantages over gravity models, including the ability to incorporate nonspatial characteristics such as capacity constraints.^26^, ^27^, ^28^ Often used for locating proposed health care facilities or emergency medical services,^29^, ^30^, ^31^, ^32^, ^33^, ^34^ optimization models can also be used to allocate demand to existing locations to estimate spatial accessibility.^35^ A constrained optimization approach involves probabilistically allocating patient populations to nearby primary care providers until the capacity for that provider is met. Any unallocated population is then assigned to the next closest provider so long as the location is within the travel distance constraints. For example, Zheng et al^36^ used optimization models to measure access to pediatric primary care in Georgia, and Gentili et al^27^ examined differences in pediatric primary care access by insurance status and rurality across seven states, finding significant disparities in accessibility in both rural and urban communities.

In this study, we examine the adequacy of the supply of all primary care providers in Iowa. In order to provide a standard for comparison, we use the same maximum travel distance and population‐to‐provider ratio thresholds that are used to identify geographic HPSAs for primary care. However, we also include NPPs to examine their impact on spatial accessibility. We compare the results of a capacitated coverage optimization model^37^ with the existing HPSAs for primary care in Iowa, considering both physicians and NPPs.

Our research questions include the following:

1. How well do the current designations for geographic HPSAs reflect spatial access to primary care physicians?
2. What is the impact of NPPs on the spatial access to primary care?
3. How do the impacts of NPPs on spatial access vary between urban and rural areas?

Iowa is a good candidate for this type of study for several reasons. First, a large proportion of Iowa counties (29/99) are at least partially covered by geographic HPSAs for primary care. This includes 11 percent of the state's population and nearly 25 percent of the land area. [An additional 20 counties are designated HPSAs for primary care for low‐income or Medicaid‐eligible populations.] While the HPSA designation is controversial,^38^, ^39^ its standards of a 30‐minute travel time and primary care physician to population ratio of 1:3500 provide benchmarks to evaluate the impact of NPPs on the adequacy of the primary care workforce in Iowa at a fine‐grained level.

Second, the state of Iowa recognizes PAs and APRNs as primary care providers and grants APRNs full independent practice authority and prescriptive authority (Iowa Code Ann. §135.157(9)). Iowa is one of only eighteen “full practice” states with the fewest restrictions on NPPs.^40^ It is important to study a state such as Iowa since researchers have suggested that one key to expanding the primary care workforce is through the liberalization of the scope of practice (SOP) for NPPs.^41^ By studying the location patterns of NPPs in Iowa, we can evaluate whether these providers choose to practice in rural areas as suggested in related research^42^, ^43^ or do they tend to cluster in locations already well‐supplied with physicians as in California,^44^ a state with more restrictions of the scope of practice for APRNs.

Finally, Iowa maintains a continuously updated roster of practicing physicians, APRNs and PAs. This provider data include practice focus (eg, hospital medicine versus urgent care) which is a key advantage for determining the actual size of the primary care workforce once NPPs are included.^19^

## 2 METHODS

### 2.1 Data

Data on the primary care provider workforce in 2017 were obtained from the Iowa Health Professions Inventory (IHPI), maintained by the Office of Statewide Clinical Education Programs, Carver College of Medicine (University of Iowa). The IHPI currently tracks all physicians, dentists, pharmacists, PAs, and APRNs practicing in the state. Provider data include demographic (birth year, sex), educational (school, graduation year), and professional (eg, specialty, worksite address) information. It is regularly updated using a number of data sources including a semiannual telephone census of all health care practice locations in Iowa.

To identify primary care providers, we followed the Institute of Medicine (IOM) definition of primary care,^45^ as operationalized by Spetz et al (2015).^19^ We included the specialties of family medicine, general internal medicine, general pediatrics, and obstetrics/gynecology. Providers working at federal health care institutions (eg, VA hospitals or clinics) and those working at urgent care clinics^19^ were excluded. Urgent care clinics are classified as non–primary care settings by Spetz et al (2015) and others, consistent with the IOM definition of primary care which specifies “a sustained partnership with patients” relating to continuity of care and ongoing relationships between provider and patient,^4^, ^19^, ^45^ factors usually lacking in urgent care settings. We also excluded providers employed in administrative, hospitalist, or nonclinical roles.

The classification of physicians was based on their self‐reported specialty. Self‐reported specialty was not available for NPPs, requiring the use of worksite as a proxy for participation in primary care. For PAs, their practice specialty was reported by the PA's worksite. For the APRNs, we first limited our sample to those trained as family nurse practitioners (NPs), pediatric NPs, adult NPs, Ob/Gyn NP, nurse midwife, and women's health NP. After imposing the restrictions listed above, we then examined the worksite of each APRN, eliminating those working for specialty medical practices, for example, cardiology, from our sample.^19^

A number of studies^41^, ^46^, ^47^, ^48^ provide estimates of the relative productivity of primary care providers, including NPPs. Following these estimates, physicians (ie, MDs and DOs) reporting full‐time status were assigned a full‐time equivalent (FTE) of 1.0, while those reporting part‐time were assigned an FTE of 0.5. Physicians employed in gynecology‐obstetrics were assigned an FTE of 0.25/0.125 for full‐time/part‐time status.^41^ PAs were assigned an FTE of 0.886/0.443 for full‐time/part‐time positions.^47^ APRNs were assigned an FTE of 0.592/0.296 for full‐time/part‐time roles.^47^

It should be noted that, unlike the HPSA scoring model for primary care, we do not have information at the individual provider level regarding their acceptance of various insurance plans including Medicaid patients. However, if such information about providers and the surrounding population were available to the analyst, it could be incorporated into the constrained optimization model.

Population data were obtained from the US Census's American Community Survey 5‐year 2017 estimates at the minor civil division (MCD) scale. MCDs are subcounty divisions used in Iowa for defining HPSA boundaries. There are more MCDs in Iowa (n = 1662) than zip codes (n = 963) or census tracts (n = 825 for 2010) allowing for a finer level of geographic precision. For comparison, analyses were also performed at the Census Block Group scale (see Appendix S1).

Information on current geographic HPSAs for primary care was obtained from HRSA. Following current guidelines for geographic HPSA designation for primary care, provider capacity was calculated as the total FTE value within each MCD multiplied by 3500, corresponding to the 3500:1 population‐to‐provider threshold. This is a simplifying assumption to the extent that we do not incorporate population‐level factors that may increase the demand for primary care services (eg, high levels of poverty and high incidence of low birthweights). This is due in part to the poor quality of these estimates at high levels of geographic specificity (compared to county‐level estimates). If reliable information were available at the subcounty geographic level of analysis (MCD, census block group, etc), it could be readily incorporated into the model formulation.

We geocoded practice locations for primary care providers using ArcMap 10.7 (Esri, CA, USA) and reference data from TomTom (Amsterdam, Netherlands) with a matching rate of 97 percent. The remaining records (n = 117) were manually rematched. The centroid of the MCD was considered the location of the patient population and any primary care providers practicing within its boundaries.

To compare the impact of NPPs on spatial access for urban and rural areas, we classified MCDs into one of 10 primary rural‐urban commuting areas (RUCA version 3.1) codes using census tract‐level data. We assigned the same designation to every MCD whose centroid fell within the tract's boundaries. We further consolidated the 10 primary RUCA codes into five levels of increasing rurality.^49^ The first level consisted of all urban core areas, that is, those contiguous areas of 50 000 or more people (RUCA code 1), and the second included densely populated suburban areas (RUCA codes 2 and 3 with a population density of 100 + per square mile). Large rural towns include populations of 10 000‐49 999 (RUCA codes 4‐6) with a population density of 100+ per square mile. Small rural towns include populations of <10 000 (RUCA codes 7‐10) and a population density between 50 and 100 per square mile. Rural areas include all locations outside the urban core areas with a population density <50 per square mile. This classification distinguishes between small rural towns that may have a population sufficient to support a primary care provider and more sparsely populated rural areas.

### 2.2 Methods

To measure potential spatial accessibility to primary care in Iowa, we created an optimization model to allocate demand (population) to capacitated supply (providers), with apportioned demand. We used ArcMap to estimate road travel times between all MCD centroids. The allocation procedure starts with population demand points located at MCD centroids. One MCD is selected at random and its population is allocated to the nearest provider until the capacity of that provider is reached or the entire population is allocated. Any unallocated population can be apportioned to the next nearest provider within the maximum travel time threshold of 30 minutes. Once demand has been allocated or all available capacity within the maximum travel time threshold has been exhausted, another MCD is selected at random and the process repeats until no further allocation can occur.

Since the results may depend upon the order in which MCDs are selected, 10 000 random permutations were run and the results summarized. In some locations, the population can never be completely allocated within the maximum travel time threshold, resulting in “confirmed shortages” that were present in every one of the 10 000 scenarios. In other MCDs, the population can sometimes be allocated, but other times cannot. These are classified as “probable” (some level of unallocated population in >50 percent of the scenarios) or “possible” (some level of unallocated population in at least 1, but <50 percent of the scenarios) shortage areas. In other locations, the population is always successfully allocated resulting in no shortage. For each of the 10 000 scenarios, the number of people who were not assigned to a primary care provider was recorded. We report the mean unallocated population for each MCD.

Models were first run considering only primary care physicians, in accordance with current HPSA geographic shortage designation criteria. Next, the models were run considering all primary care providers, both physicians and NPPs. Allocation analyses were performed in R v3.5.0 (R Foundation for Statistical Computing). Maps were created using ArcGIS Pro 2.2.4 (ESRI).

## 3 RESULTS

In 2017, there were 2424 Iowa physicians with a specialty in primary care. Of these, 2000 were providing primary care under the operational definition described above with a total FTE of 1781.63. Considering the state as a whole, the population‐to‐primary care FTE ratio was 1744:1. This is approximately double the 3500:1 ratio used in the determination of geographic HPSAs for primary care suggesting that, at the state level, there is no shortage of primary care physicians.

A total of 517 PAs were practicing under the supervision of physicians with a specialization in primary care. After filtering, the number of PAs in primary care was 423 with a total FTE of 358.4. There were 1681 APRNs with training in primary care fields. Of these, 1114 were practicing in primary care in 2017. The total FTE for APRNs was 633.7.

In 2017, there were 3537 primary care providers including both physicians and NPPs, with a total FTE of 2773.7. Across the entire state, this would give a population: FTE ratio of 1120:1.

### 3.1 Spatial accessibility

One‐quarter (408/1662) of MCDs were located in geographic HPSAs for primary care. These MCDs contained approximately 11 percent (n = 349 251/3 106 589) of the state's population. Only 5.1 percent [101/2000] of primary care physicians practiced in geographic HPSAs. An additional 316 MCDs were in areas classified as population (Medicaid‐eligible or low‐income) HPSAs for primary care. These MCDs include an additional 16 percent (n = 486 170/3 106 589) of the state's population.

Based on the constrained optimization model described above, a total of 361 MCDs (22 percent) were confirmed shortage areas for primary care physicians for all 10 000 scenarios (Table 1). These 361 confirmed shortage areas together contained only one primary care physician. Only 109 (30 percent) of these MCDs were within existing geographic HPSAs. The majority (187/361 or 52 percent) of the MCDs with confirmed shortages were located outside a designated HPSAs (either geographic or population). We also note that nearly half (194/408 or 48 percent) of the MCDs within a geographic HPSA had no shortage based on the 10 000 scenarios.

**Table 1:** Mean unallocated population and number of minor civil divisions (MCDs) with primary care shortages for physicians only and for physicians and nonphysician providers (NPPs), stratified by location within or outside current health professional shortage areas (HPSAs)

	Physicians only		Physicians + NPPs	
	Mean unallocated population	MCDs	Mean unallocated population	MCDs
In geographic HPSAs				
Confirmed shortages (100% of runs)	66 935	109	17 158	36
Probable shortages (≥50% of runs)	8641	23	2312	11
Possible shortages (<50% of runs)	12 815	82	1206	19
No shortage	0	194	0	342
In population HPSAs				
Confirmed shortages (100% of runs)	23 862	65	11 923	40
Probable shortages (≥50% of runs)	4839	19	326	3
Possible shortages (<50% of runs)	529	7	277	4
No shortage	0	225	0	269
Outside any HPSAs				
Confirmed shortages (100% of runs)	90 287	187	39 451	94
Probable shortages (≥50% of runs)	5934	14	2868	15
Possible shortages (<50% of runs)	6660	59	2730	35
No shortage	0	678	0	794
Entire state				
Confirmed shortages (100% of runs)	181 084	361	68 532	170
Probable shortages (≥50% of runs)	19 414	56	5506	29
Possible shortages (<50% of runs)	20 004	148	4213	58
No shortage	0	1097	0	1405

The average unallocated population in the MCDs with a confirmed shortage is substantial, representing 6 percent (181 084/3 118 102) of the state's population. These MCDs represent 82 percent (181 084/220 502) of the expected shortfall in spatial access to primary care physicians.

Turning to the addition of NPPs, we find that 8.6 percent (270/3156) of primary care providers practiced in geographic HPSAs in 2017. The additional 170 FTE of primary care represents an increase of 170 percent in these 408 MCDs. Within geographic HPSAs, the number of MCDs with a confirmed shortage fell by 67 percent (from 109 to 36). The comparable figure for population HPSAs was 38 percent and for areas outside of a designated HPSA, the reduction was 50 percent (from 187 to 94).

When NPPs were included in the measure of spatial accessibility, the level of unallocated population in the 361 MCDs with a confirmed shortage fell by 62 percent (from 181 084 to 68 532). Within geographic HPSAs, the reduction was greater at 74 percent (from 66 935 to 17 158).

**Figure 1:**
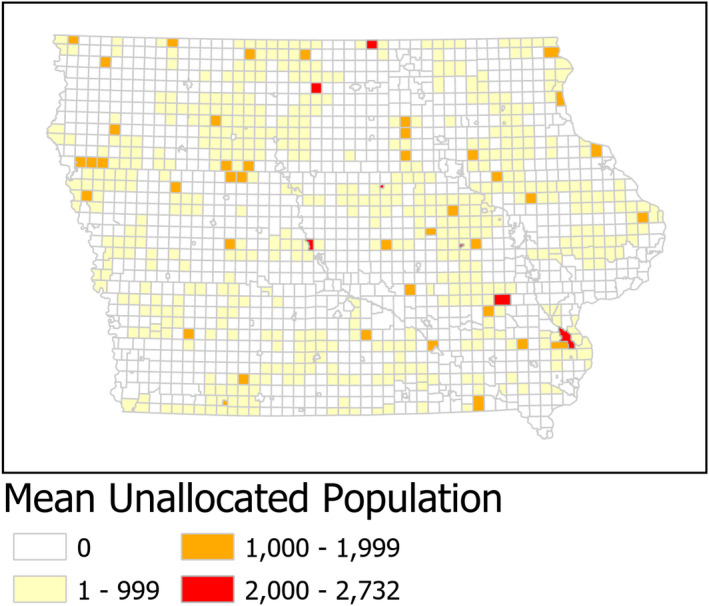
Mean unallocated population for primary care using primary care physicians (n = 10 000 scenarios) [Colour figure can be viewed at wileyonlinelibrary.com]

The prior analysis focused on MCDs with a confirmed shortage. However, there are MCDs for which some but not all of the people are unassigned to a provider within a 30‐minute drive. The exact number for each MCD varied based on the probabilistic assignment scheme. Therefore, we average the results across the 10 000 scenarios. Based on the location and number of primary care physicians, the average unallocated population for each MCD is summarized in Figure 1.

**Figure 2:**
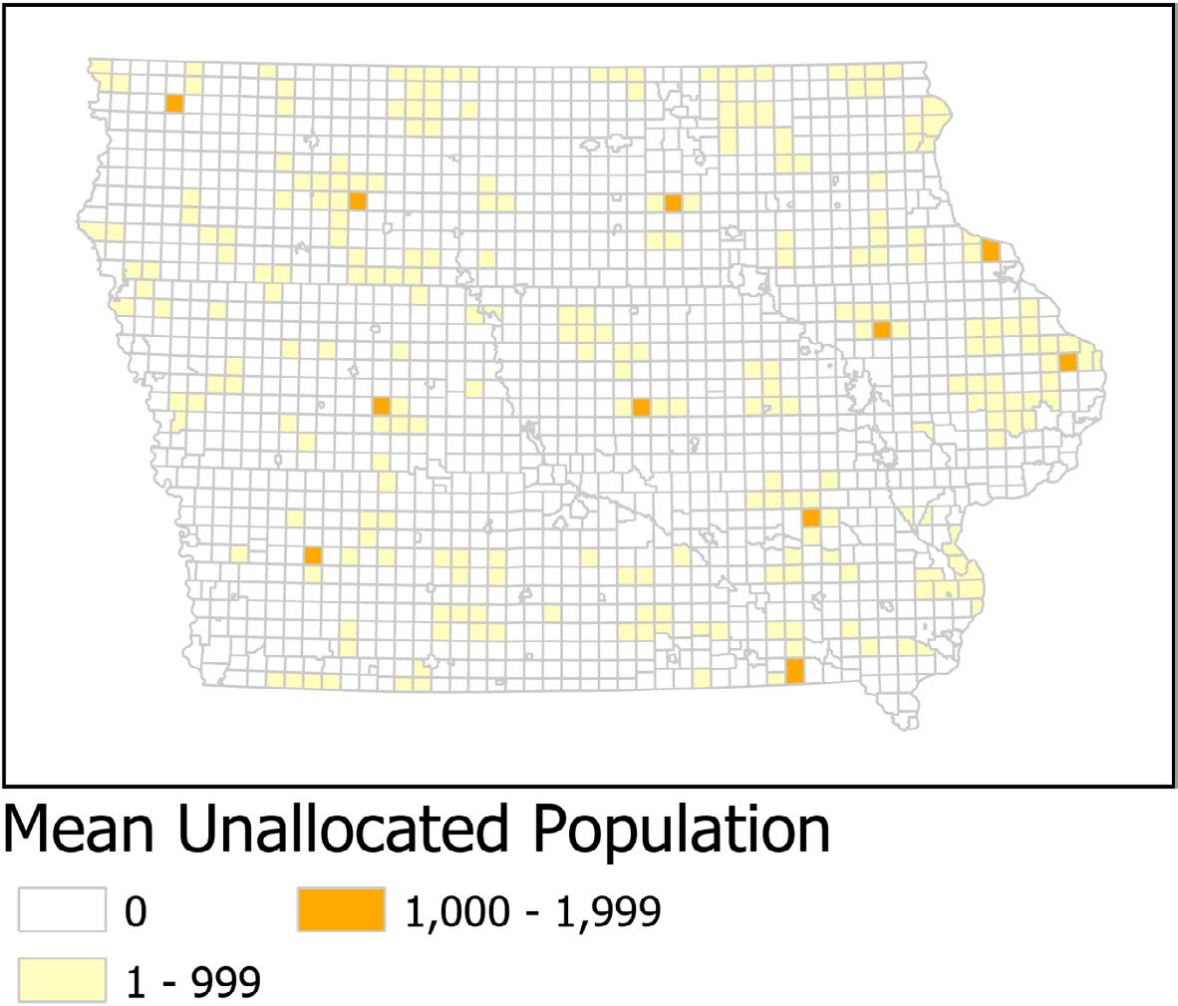
Mean unallocated population for primary care using both primary care physicians and nonphysician providers [Colour figure can be viewed at wileyonlinelibrary.com]

Considering all MCDs, the addition of NPPs reduced the average unallocated population from 220 502 (or 7 percent of Iowa's population) to 78 252 (or 2.5 percent of the state's population). This represents a 65 percent reduction. The remaining average unallocated population at the MCD level is mapped in Figure 2.

### 3.2 Comparison of rural and urban areas

In Table 2, we examine the impact of NPPs on the supply of primary care providers in urban and rural areas of Iowa. We also evaluate the impact of NPPs on spatial accessibility to primary care across different types of urban and rural areas. We then aggregated all measures across all 1662 MCDs for the five types of locations discussed above (urban core, suburbs, large rural town, small rural town, and rural).

**Table 2:** Distribution of population, MCDs, mean unallocated population for primary care, and full‐time equivalents (FTE) for primary care physicians and NPPs, stratified by rurality of location

	2017 Population (% of total)	MCDs	Average population per MCD	Mean unallocated population—physicians only (% of population)	Mean unallocated population—physicians + NPPs (% of population)	Primary care FTE—physicians only (% of total)	Primary care FTE—physicians + NPPs (% of total)	Change in primary care FTE with NPPs (% of total)
Urban core	1 349 111 (43%)	62	21 760	1066 (<1%)	11 (<1%)	956 (54%)	1406 (51%)	450 (45%)
Suburbs	210 042 (7%)	35	6001	3655 (2%)	0 (0%)	129 (7%)	175 (6%)	46 (5%)
Large rural town	298 404 (10%)	23	12 974	0 (0%)	0 (0%)	205 (11%)	307 (11%)	102 (10%)
Small rural town	539 884 (17%)	155	3483	26 524 (5%)	359 (<1%)	381 (21%)	646 (23%)	265 (27%)
Rural area	720 661 (23%)	1387	520	189 256 (26%)	77 882 (11%)	112 (6%)	240 (9%)	128 (13%)
Total	3 118 102	1662	1876	220 501 (7%)	78 252 (2.5%)	1783	2774	991

Some 60 percent of Iowans live in an urban core, suburban, or high‐density (> 100/square mile) large rural towns. The average population per MCD for small rural towns and rural areas are a small fraction of those observed in more densely populated areas.

As expected, areas in the urban core have a higher proportion of the primary care physicians than their population (54 percent of FTE versus 43 percent of population). The same is true, to a lesser extent in large rural towns (11 percent of primary care physician FTE compared to 10 percent of the population). Suburbs have a comparable proportion of primary care FTE (7 percent) compared to their population (7 percent). Small town MCDs have a higher than proportionate share of primary care physicians (21 percent of FTE versus 17 percent of population). Rural areas with 23 percent of the population have only 6 percent of FTE primary care physicians.

Accounting for location, we see that most (86 percent) of the expected unallocated population for primary care is associated with rural areas, where more than a quarter of the population is unallocated. Most of the remaining unallocated population for primary care is found in small rural towns (12 percent). It is interesting to note that large rural towns have no unallocated population. This may be due to the large number of hospitals in these rural cities across Iowa.

With the addition of NPPs, the capacity for providing primary care rises across all categories of location. However, the change is not uniform. Urban core areas capture 45 percent of the increase in FTEs followed by small rural towns with an increase of 27 percent. Rural areas account for 13 percent, large rural towns 10 percent, and suburbs account for 4.6 percent of the increase in FTEs. Proportionally, rural areas see an increase in FTEs of 114 percent, followed by small rural towns (70 percent), large rural towns (50 percent), urban core areas (47 percent), and suburban areas (36 percent).

With the addition of the NPPs, the unallocated population in the urban core areas is reduced to a fraction of 1 percent. The largest change in unallocated population is in rural areas. Fully 78 percent of the reduction in unallocated population occurs in rural areas. An additional 18 percent of the reduction in unallocated population occurs in small rural towns. Consequently, almost all (96 percent) of the positive effects of NPPs on spatial accessibility are associated with rural areas of Iowa.

## 4 DISCUSSION

Our analysis shows that many MCDs in Iowa with sufficient spatial access to primary care are included in geographic HPSAs while, at the same time, many MCDs without sufficient access are excluded from any shortage designation whatsoever. Such errors suggest that the methods used for identifying geographic HPSAs should be modernized to take advantage of state‐of‐the‐art developments in geospatial analysis.

We further show that the impact of NPPs on spatial access to primary care is considerable. The number of individual MCDs with no spatial access to primary care fell more than 50 percent. At the same time, the number of MCDs with no shortage (across 10 000 scenarios) rose from 66 to 85 percent. At the state level, the addition of NPPs to the primary care workforce resulted in a reduction in the overall unallocated population by more than 60 percent.

Like its primary care physicians, most of Iowa's NPPs are located in densely populated urban areas. Forty percent of the NPPs were located in small rural towns and sparsely populated rural areas where almost all of the shortfall in spatial access to primary care lies. However, the NPPs choosing to practice in these low‐density rural areas have a profound impact on spatial access. In these areas, the percentage of the unallocated population is 17 percent when the primary care workforce is limited to physicians. This figure falls to 6 percent when the contributions of NPPs are recognized.

**Figure 3:**
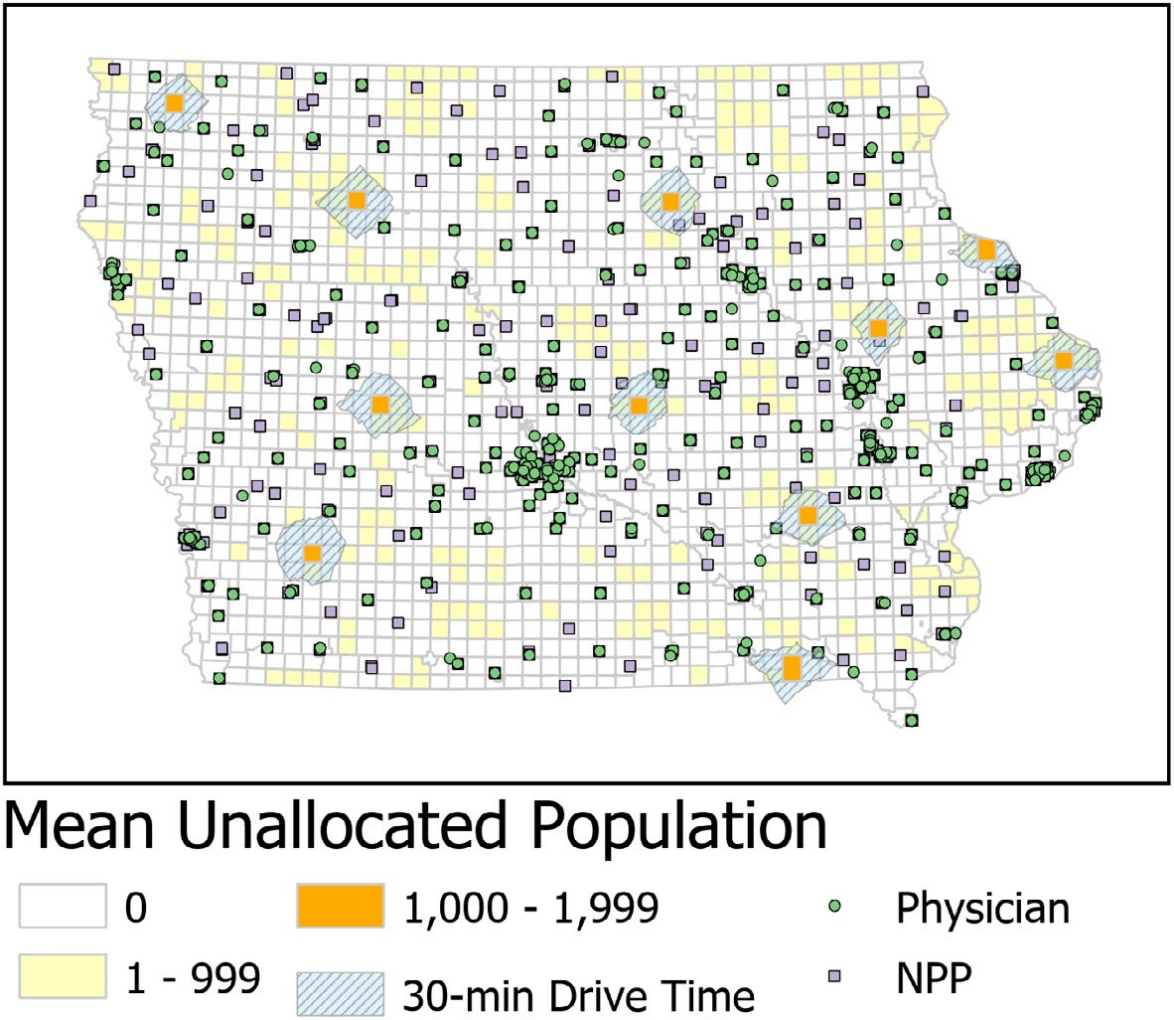
Mean unallocated population for primary care using both primary care physicians and nonphysician providers, with provider locations and 30‐min driving distance polygons for areas of greatest unallocated population indicated [Colour figure can be viewed at wileyonlinelibrary.com]

While the addition of NPPs reduces the unallocated population for primary care, there remain shortage areas across the state. In Figure 3, we combine information about current locations of all primary care providers along with 30‐minute driving distances from the 11 MCDs with the largest unallocated population. For some of these populations, there are existing primary care providers within a 30‐minute drive. Meeting the needs of these patients would require additional practitioners in existing facilities. For other locations, the challenge is greater since there is no existing provider within a 30‐minute drive. To meet the needs of these patients, there would have to be investment in a new facility to facilitate primary care providers. This is likely to represent a greater financial hurdle than adding staffing to an existing location. In either case, using the spatial accessibility approach described in this study, health officials can better use their limited resources in a targeted way to improve access to primary care.

### 4.1 Limitations and future directions

Since this study focuses on a single state, it has limitations to its generalizability. The observed improvement in access for rural populations may not be relevant in more highly urbanized states, although the optimization models used are appropriate for such areas. Iowa may also have “edge effects” near the state's borders, although it is difficult to estimate the magnitude since errors flow in both directions across state lines.^50^ Future studies should include border areas of neighboring states when possible to determine whether discontinuities are artifacts or authentic. This would require a regional system to track health professionals (and their practice specialties) at a highly disaggregate level.

Rural states are not homogeneous. Geographic, regulatory, and other differences may result in a lower or higher measured impact of NPPs on spatial access to primary care. For instance, state‐level scope‐of‐practice laws influence what primary care services NPPs can offer; thus, states with more restrictive scope can expect less of an improvement in access than was observed in Iowa.

Our study focused only on spatial access to primary care. There remain many other demographic, cultural, and financial barriers for patients to overcome in order to realize the promise of equitable access to primary care. Other nonpatient factors, for example, low levels of Medicaid reimbursement or transportation challenges,^51^, ^52^, ^53^ may likewise affect realized access to primary care services and should be included in future studies.

## 5 CONCLUSION

Our analysis of Iowa's primary care workforce suggests that the crisis surrounding primary care physicians could be viewed as a spatial demand‐capacity mismatch.^54^ At the state level of geography, there is no shortage of primary care physicians but they are unevenly allocated, leading to localized areas of poor or no spatial accessibility.

There are substantial funding implications for geographic HPSA designations. However, the current system only considers the role of physicians in providing primary care. Consistent with research from other countries, we find that the addition of NPPs to the primary care workforce results in a considerable reduction in the population without sufficient spatial access to primary care. Almost all of the improvement occurs in sparsely populated rural areas, which are also the areas of greatest need. In an era of needs growing faster than budgets, more accurately measuring the needs of rural populations should result in better uses of limited funds.

As a case study, these results from Iowa show how high‐quality data on NPP location, training, and practice settings can be leveraged using microlevel models of demand for primary care. Our single measure of spatial accessibility—generated using a probabilistic, constrained optimization algorithm run over 10 000 scenarios—identified areas of inaccessibility to primary care within and outside of areas designated as HPSAs for primary care. Including the presence of primary care NPPs, we are able to quantify their considerable contributions to reducing the unallocated population and characterize their effect on patients in underserved rural locations. The approach presented in this paper could help policy makers better target resources dedicated to increasing access to primary care services to those areas of truly greatest need.

## CONFLICT OF INTEREST

The authors report no other disclosures.
