# Supplementary material for: Impact of nonphysician providers on spatial accessibility to primary care in Iowa
Source: Health Serv Res. 2020 Feb 26;55(3):476–85. doi: 10.1111/1475-6773.13280 (PMC7240764; doi:10.1111/1475-6773.13280)

# Supplement to Impact of non-physician providers on spatial accessibility to primary care in Iowa

For comparison with our findings at the Minor Civil Division scale, we also performed the allocation analyses using Census Block Groups (CBGs) and found broadly similar trends. Below are the results of these analyses.

*Table S1: Mean unmet demand and number of Census block groups (CBGs) with primary care shortages for physicians only and for physicians and non-physician providers (NPPs)*, stratified by location within or outside current Health Professional Shortage Areas (HPSAs).

|  | | Physicians Only | | Physicians + NPPs | |
| --- | --- | --- | --- | --- | --- |
|  |  | Mean Unmet Demand | CBGs | Mean Unmet Demand | CBGs |
| In Geographic HPSAs | Confirmed Shortages (100% of runs) | 55,124 | 68 | 19,480 | 23 |
|  | Probable Shortages (≥50% of runs) | 9,137 | 17 | 1,560 | 4 |
|  | Possible Shortages (<50% of runs) | 14,400 | 89 | 976 | 6 |
|  | No Shortage | 0 | 188 | 0 | 329 |
| In Population HPSAs | Confirmed Shortages (100% of runs) | 24,541 | 26 | 12,097 | 14 |
|  | Probable Shortages (≥50% of runs) | 2,748 | 5 | 2,613 | 5 |
|  | Possible Shortages (<50% of runs) | 6,193 | 21 | 943 | 5 |
|  | No Shortage | 0 | 379 | 0 | 407 |
| Outside Any HPSAs | Confirmed Shortages (100% of runs) | 86,849 | 100 | 39,417 | 46 |
|  | Probable Shortages (≥50% of runs) | 14,212 | 25 | 8,885 | 18 |
|  | Possible Shortages (<50% of runs) | 5,934 | 59 | 2,284 | 19 |
|  | No Shortage | 0 | 1,653 | 0 | 1,754 |
| Entire State | Confirmed Shortages (100% of runs) | 166,514 | 194 | 70,994 | 83 |
|  | Probable Shortages (≥50% of runs) | 26,097 | 47 | 13,058 | 27 |
|  | Possible Shortages (<50% of runs) | 26,527 | 169 | 4,203 | 30 |
|  | No Shortage | 0 | 2,220 | 0 | 2,490 |

*Table S2: Distribution of population, CBGs, mean unmet demand for primary care, and full-time equivalents (FTE) for primary care physicians and NPPs, stratified by rurality of location.*

|  | 2017 Population  (% of Total) | CBGs | Average Population per CBG | Mean Unmet Demand - Physicians Only  (% of Population) | Mean Unmet Demand – Physicians + NPPs  (% of Population) | Primary Care FTE - Physicians Only  (% of Total) | Primary Care FTE - Physicians + NPPs  (% of Total) | Change in Primary Care FTE With NPPs  (% of Total) |
| --- | --- | --- | --- | --- | --- | --- | --- | --- |
| Urban Core | 1,454,810  (45%) | 971 | 1,498 | 0  (0%) | 0  (0%) | 1029  (58%) | 1495  (54%) | 466  (47%) |
| Suburbs | 166,989  (5%) | 111 | 1,504 | 7,526  (5%) | 2,732  (2%) | 58  (3%) | 91  (3%) | 33  (3%) |
| Large Rural Town | 323,848  (10%) | 296 | 1,094 | 4,038  (1%) | 2,821  (1%) | 232  (13%) | 341  (12%) | 109  (11%) |
| Small Rural Town | 572,344  (x%) | 531 | 1,078 | 45,244  (8%) | 6,730  (1%) | 380  (21%) | 663  (24%) | 283  (29%) |
| Rural Area | 681,557  (x%) | 721 | 945 | 162,331  (24%) | 75,973  (11%) | 84  (5%) | 184  (7%) | 100  (10%) |
| Total | 3,199,548 | 2,630 | 1,217 | 219,138  (7%) | 88,256  (3%) | 1,783 | 2,774 | 991 |

*Figure S1: Mean unallocated population for primary care using primary care physicians (n = 10,000 scenarios).*


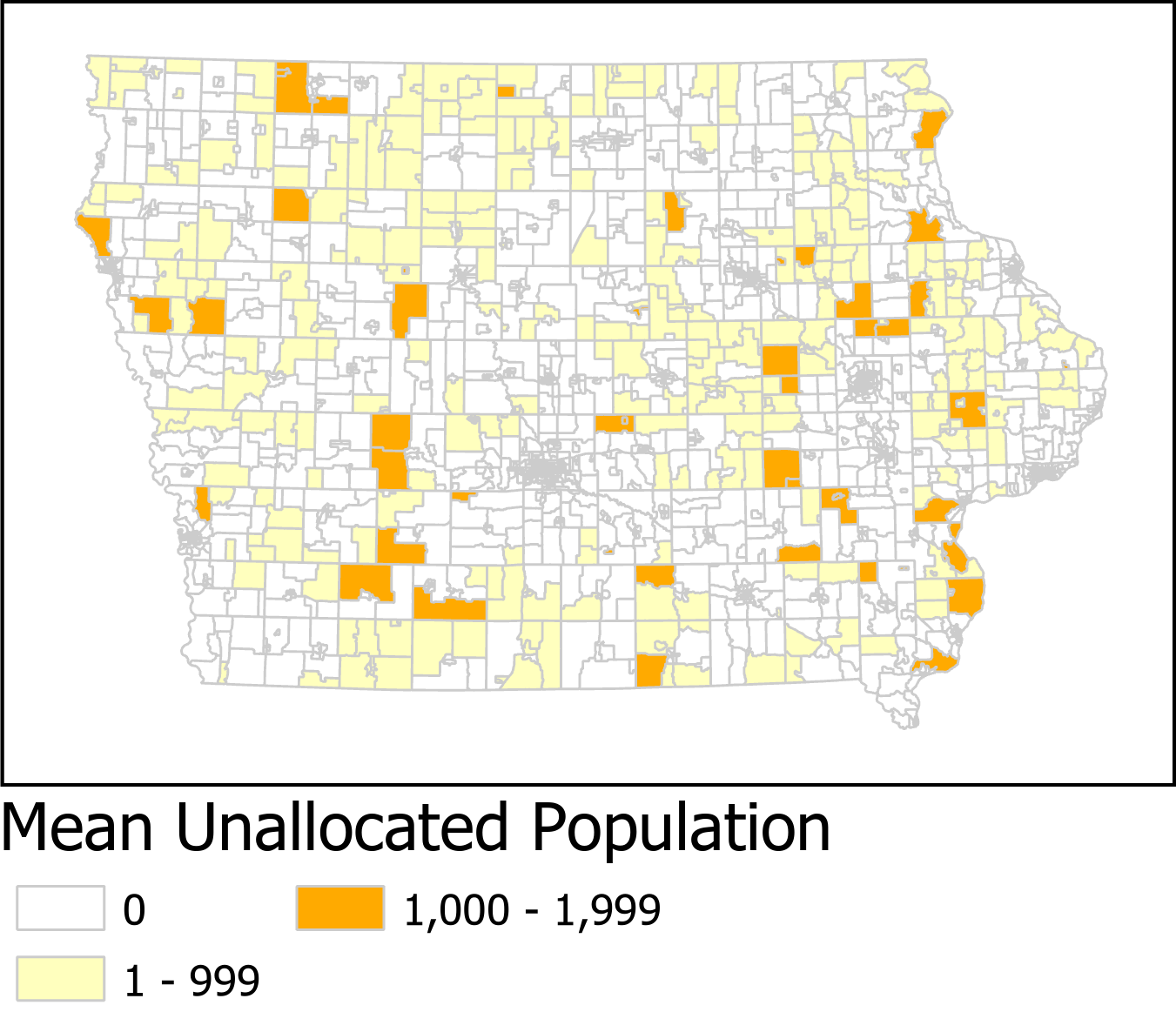


*Figure S2: Mean unallocated population for primary care using both primary care physicians and NPPs.*


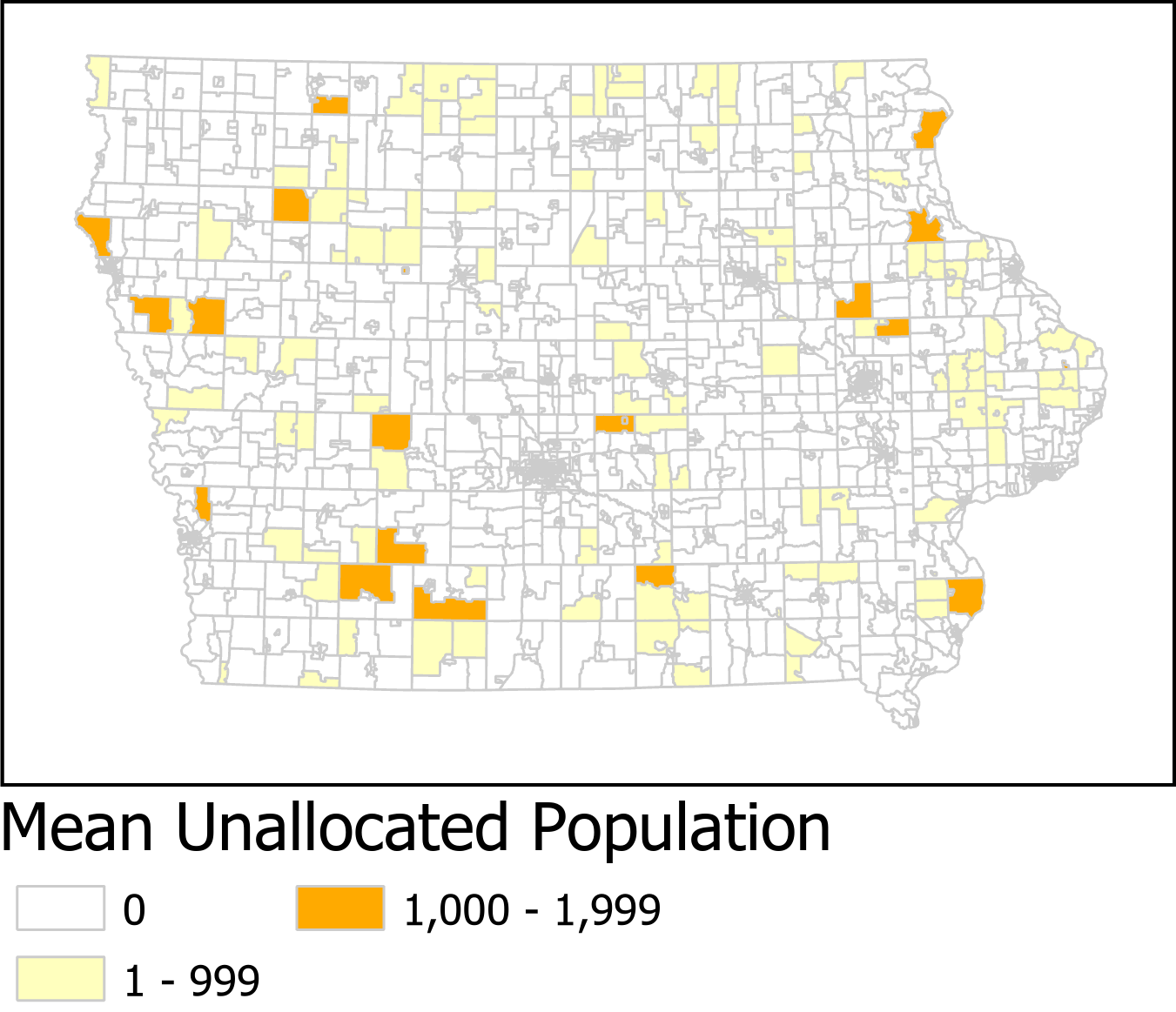

Supplement: Supplementary file 2 [file HESR-55-476-s002.doc]
